# Trabecular Meshwork Gene Expression after Selective Laser Trabeculoplasty

**DOI:** 10.1371/journal.pone.0020110

**Published:** 2011-07-01

**Authors:** Alberto Izzotti, Mariagrazia Longobardi, Cristina Cartiglia, Federico Rathschuler, Sergio Claudio Saccà

**Affiliations:** 1 Department of Health Sciences, Faculty of Medicine, University of Genoa, Genoa, Italy; 2 Ophthalmology Unit, Department of Head/Neck Pathologies, St. Martino Hospital, Genoa, Italy; Kyushu Institute of Technology, Japan

## Abstract

**Background:**

Trabecular meshwork and Schlemm's canal are the tissues appointed to modulate the aqueous humour outflow from the anterior chamber. The impairment of their functions drives to an intraocular pressure increase. The selective laser trabeculoplasty is a laser therapy of the trabecular meshwork able to decrease intraocular pressure. The exact response mechanism to this treatment has not been clearly delineated yet. The herein presented study is aimed at studying the gene expression changes induced in trabecular meshwork cells by selective laser trabeculoplasty (SLT) in order to better understand the mechanisms subtending its efficacy.

**Methodology/Principal Findings:**

Primary human trabecular meshwork cells cultured in fibroblast medium underwent selective laser trabeculoplasty treatment. RNA was extracted from a pool of cells 30 minutes after treatment while the remaining cells were further cultured and RNA was extracted respectively 2 and 6 hours after treatment. Control cells stored in incubator in absence of SLT treatment were used as reference samples. Gene expression was evaluated by hybridization on miRNA-microarray and laser scanner analysis. Scanning electron microscopic examination was performed on 2 Trabecular meshwork samples after SLT at 4^th^ and 6^th^ hour from treatment. On the whole, selective laser trabeculoplasty modulates in trabecular meshwork the expression of genes involved in cell motility, intercellular connections, extracellular matrix production, protein repair, DNA repair, membrane repair, reactive oxygen species production, glutamate toxicity, antioxidant activities, and inflammation.

**Conclusions/Significance:**

SLT did not induce any phenotypic alteration in TM samples. TM is a complex tissue possessing a great variety of function pivotal for the active regulation of aqueous humour outflow from the anterior chamber. SLT is able to modulate these functions at the postgenomic molecular level without inducing damage either at molecular or phenotypic levels.

## Introduction

**Figure 1:**
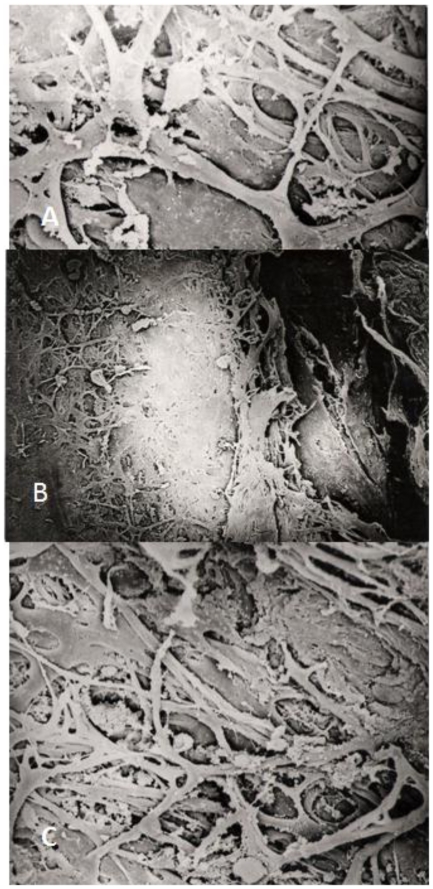
Trabecular meschwork after SLT and ALT. Scanning electron microscope photograph of the human sclerocorneal trabecular meshwork (magnification 2,000x in A and C, and 1,500x in B) 3 h (A) and 6 h (C) after SLT treatment. The architecture of TM is well conserved, showing intact trabecular beams (A,C). By comparison, following argon laser trabeculoplasty a coagulative damage with disruption of trabecular beams is well evident (B).

Glaucoma is a neurodegenerative multi-factorial disease affecting different target tissues: the lateral geniculate nucleus and the visual cortex in the central nervous system, [1] the optic nerve head in the retina, and the trabecular meshwork (TM) in the anterior chamber (AC) of the eye. In the majority of cases the glaucoma is accompanied by intraocular pressure increase that is the most important risk factor for the progression of disease [2]. All the pathogenic events leading to death of the retinal ganglion cells (RGCs) are not yet known with accuracy but it is established that TM play a major role in the glaucomatous pathogenic cascade. The concept that eye outflow system is a passive filter is outdated. Indeed Alvarado found that severe alterations occur in the cellular component and in the entire TM during POAG and ageing [3], [4]. The same author shown that TM endothelial cells regulate aqueous outflow by actively releasing enzymes and cytokines that, upon binding to Schlemm's canal (SC) endothelial cells, increase transendothelial flow thereby facilitating the egress of aqueous humour [5]. TM endothelial cells secrete these factors in response to stimuli such as mechanical stretching, laser irradiation, and pro-inflammatory cytokines [6], [7]. In 1991 Saccà et al. [8], [9] presented a new Argon Laser Trabeculoplasty (ALT) technique as applied to TM that took advantage of a very low power thus not creating burns in TM and being at the same time able to obtain a lasting effect of IOP decrease in subjects suffering from glaucoma (Figure 1). Some years later Latina et al. [10] employed a q-switched 532-nm neodymium (Nd):YAG laser to investigate the safety and efficacy of laser treatment. Laser parameters were set to selectively target pigmented TM cells without coagulative damage to the TM structure or nonpigmented cells. This technique was called “selective laser trabeculoplasty,” (SLT) and decreased intraocular pressure by an amount similar to that achieved with standard trabeculoplasty [11]. Actually, SLT does not produce any anatomic alteration appreciable to microscope while ALT cause visible burns on TM (Figure 1). Anyway, SLT is equivalent to ALT in terms of IOP lowering at 1 year and is a safe and effective procedure for patients with open-angle glaucoma [12]. Indeed, the exact response mechanisms to this treatment has not been clearly delineated yet, even if it is known that energy laser has many biological effects on TM depending on the magnitude of the energy used and the distance from the center of the irradiated zone [13]. One biologic response of the trabecular meshwork after laser trabeculoplasty is a change in the level of ongoing trabecular cell division [14]. This is the basis of the ‘‘repopulation theory’’ stating that laser would stimulate the repopulation of the meshwork with fresh trabecular cells, which may result in the formation of healthy TM [15]. The “biological theory” suggests that the mechanism of action of ALT or SLT is based on the release of cytokines and synthesis of matrix metalloprotease enzyme from TM cells induced by the heating effect of the laser and finally resulting in an increased turnover of the extracellular matrix [16], [17].

The first “mechanical theory” stating that the laser application results in the local shrinkage of the meshwork, opening the intertrabecular spaces between the laser sites [18] has been nowadays neglected by most of the authors.

SLT applied on rabbit eyes increases in aqueous humour lipid peroxidase and free oxygen radicals levels [19]. Accordingly, it is conceivable that SLT induces a transient and moderate cell stress triggering cellular defences without coagulative damage to the TM [20]. In order to explore this hypothesis and gene-expression changes preceeding SLT effects, we performed the herein presented study aimed at studying the gene expression changes induced in TM cells by SLT.

The study adhered to the tenets of the Declaration of Helsinki and was approved by the Ethical Board of the Ophthalmologic Division.

## Results

SLT did not induce any phenotypic alteration in TM samples collected from corneal donors as evaluated by electron scanning microscopy either 3 h and 6 h after treatment (Fig. 1 a and c). By comparison, this lack of phenotypic effect is completely different from the remarkable tissue alterations induced by trabeculoplasty treatment in analogue sample after 3 h since treatment (Fig. 1 b). Conversely, SLT induced important changing at postgenomic level in TM cells.

**Figure 2:**
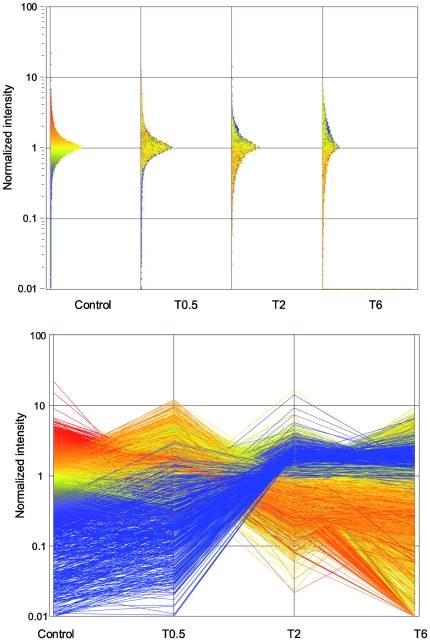
Quantitative analysis of TM Gene expression after SLT. Box plot (upper panel) and time course (lower panel) analyses of the expression of 18,401 genes in TM cells either unexposed (Control) or after various times (0.5, 2, and 6 hours) since SLT treatment. Box plot analysis report the level of expression of each gene (dot) located on vertical axis according to its level of expression expressed on a color scale referred to control (blue low expression, red high expression). Time course analysis reports the variation in the gene expression intensity for each analysed gene. Genes undergoing downregulation after SLT treatment as compared to control are indicated in blue, genes undergoing upregulation in red.

**Figure 3:**
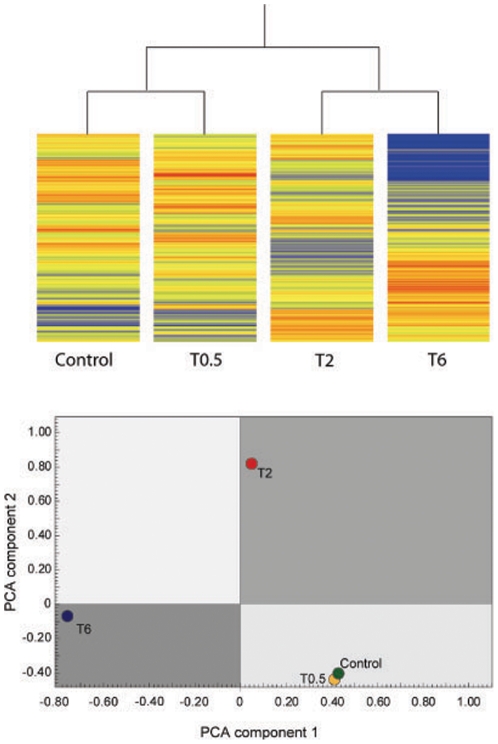
Qualitative analysis of TM Gene expression before and after SLT. Hierarchical cluster (upper panel) reporting gene expression profile as evaluated in TM cells before SLT (Control, left column) and at various times after SLT (0.5, 2, 6 h). Gene expression intensity is reported as color scale (blue low, red high). The same gene expression profile was compared among different experimental conditions (dots) by analyzing the two principal components of variance for each sample (PCA) (lower panel).

SLT treatment induced a time-related change in the gene expression profile of TM cells. As evaluated by scatter plot such a change was well detectable starting since two hours after SLT treatment and persisted up to 6 hours (Fig. 2, upper panel). Changes of gene expression induced by SLT were time dependent, being mainly detectable after 2 and 6 hours since SLT exposure, and included both upregulation of genes expressed at low levels in Control (blue lines) and dowregulation of genes expressed at high level in Control (red lines) (Fig. 2, lower panel). The modulating effect on the global gene expression profile was pointed out by hierarchical cluster analysis demonstrating that gene expression profile of Control and SLT-treated TM cells after 30 min are similar and clustered together in the same dendrogram branch (Fig. 2, upper panel, left side). Conversely, gene expression profile of SLT-treated TM cells after 2 and 6 hours are remarkably different from Control being located in different branches of the dendrogram (Fig. 3, upper panel, right side). A similar situation was recorded by performing Principal Component Analysis of variance where Control and SLT-treated TM cells after 30 min (T0.5) are located in the same quadrant, while SLT-treated TM cells after 2 hours (T2) and 6 hours (T6) are located in different quadrants far away from Control (Fig. 3, lower panel).

**Figure 4:**
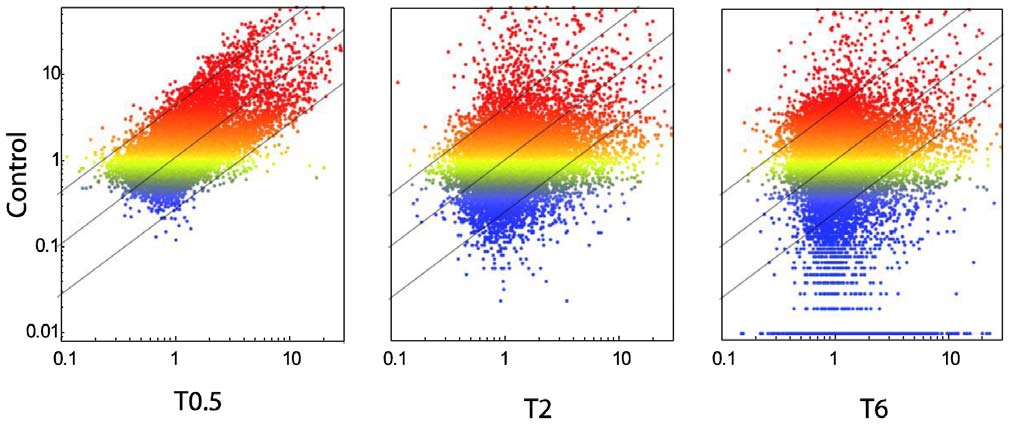
Comparative analysis of TM Gene expression before and after SLT. Scatter plot analysis comparing gene expression before (vertical axis, Control) and after SLT at various times (horizontal axis, 0.2, 2, 6 h). Gene expression intensity is reported as color scale referring to Control (blue low, red high). The diagonal lines indicate the 2-fold variation interval including genes whose expression did not change after SLT. Genes falling above these lines were downregulated, genes falling below these lines were upregulated more than 2-fold in their expression by SLT.

Scatter plot analysis identified those genes up- or down-regulated by SLT after 30 min (T0.5), 2 hours (T2) and 6 hours (T6) (Fig. 4).

No effect was observed after 30 min since SLT treatment. Conversely, after 2 hours the number of genes changing their expression more than 2-fold as compared to controls accounted for 513, further rising up to 3,774 after 6 hours.

Out of the 94 genes spotted on the microarray activating cell death by apoptosis or necrosis no one significantly varied its expression as consequence of SLT. This finding demonstrates that, under our experimental conditions, SLT does not induce directly *per se* cell damage. This finding is in line with the lack of any alteration in TM after 24 h since SLT treatment as ob served by electron microscopy (Fig. 1).

Among the many genes changing their expression, statistical support vector machine analysis identified 67 predictor genes specifically and significantly related with SLT treatment.

These genes, detailed in Table S1, online supporting information, are involved in various cell functions including cell motility, tissue integrity, cell membrane components, mitochondrion function, oxidative stress, DNA repair, glutamate metabolism, inflammation, and energy production.

Complete microarray data are available at GEO database (GEO number GSE29697) (http://www.ncbi.nlm.nih.gov/geo/).

## Discussion

Our work provides evidence at postgenomic level that TM actively modulates the aqueous humor outflow through a variety of molecular mechanisms that can be modulated by SLT.

### TM motility

Of the 67 genes modulated by SLT 7 (12%), encoding for activities involved in cell motility and contraction were upregulated by SLT indicating that this treatment enhance the contracting capacity of TM cells. The contractile function, cell shape, and cell adhesion properties of TM and SC cells have been implicated widely in modulation of aqueous humor outflow through the conventional pathway [21], [22]. Indeed, contraction of TM with its smooth muscle-like properties, decreases outflow, whereas relaxation increases this parameter [23]. This tissue has the ability to contract when exposed to appropriate stimulants [23], its property is important in helping regulate the outflow of aqueous humor [24]. Indeed, Trabecular meshwork cells express contractile elements such as smooth muscle α-actin and myosin [25]. F-actin architecture in human outflow pathway cells in situ differs between normal and glaucoma eyes, glaucomatous tissue showing a more “disordered” actin architecture overall [26]. TM and its endothelial cells, as well as SC Schlemm‘s canal and the lining endothelial cells, undergo deformation and stretching with changes in intraocular pressure [5].

The above reported results could confirm the hypothesis of the “mechanical theory” of Wise and Witter [18], neverthenless also the “biological theory” [16], [17] and the “repopulation theory” [15] are almost supported by our findings.

### Tissue integrity

Indeed, 15 out of the 67 genes (22%) modulated by SLT encoded for activities involved in maintaining TM tissue integrity. Among these, genes involved in adherence to basal membrane and intercellular connection and in extracellular matrix removal resulted up regulated. Conversely, SLT downregulated gene involved in the production of extracellular matrix. These mechanisms contributes to the maintenance of TM cellularity counteracting its decrease. It was demonstrated that a relative increase in the extracellular matrix/cells ratio is crucial for POAG progression [27]. IOP homeostasis, triggered by pressure changes or mechanical stretching of the TM, appears to involve the extracellular matrix turnover [28]. An upregulation of genes involved in repairing and removing damaged protein was observed. This finding indicate that SLT increase removal of oxidized proteins typically accumulating in degenerating and aged tissues [29].

### Cell membrane

Furthermore 8 out of 67 genes whose expression was affected by SLT treatment are cell membrane components. Genes encoding for activities involved in intracellular Ca^++^ homeostasis were modulated by SLT. This finding probably reflects the SLT effect on mitochondrion (see below), which is the main intracellular Ca^++^ depot. Intracellular Ca^++^ release is a pivotal step of the intrinsic apoptotic pathway. Thus this situation may be interpreted as a decreased trend towards apoptosis.

A study [30], examined the levels of Ca2+ in TM cells from POAG and nondiseased aged-matched individuals and demonstrated that both [Ca2+]c and [Ca2+]m were higher in POAG TM. POAG TM cells have defective mitochondrial function, which causes them to be abnormally vulnerable to Ca^2+^ stress. The dysfunction in calcium regulation by these cells may contribute to the failure of this tissue to control IOP.

SLT treatment increased the expression of genes encoding for cell growth factor, aminoacids, and Na^+^ uptake. On the whole these genes contribute to tissue integrity by stimulating cell growth and volume maintenance. SLC3A2, involved in the synthesis of nitric oxide, was upregulated. This indicates that SLT activates mechanisms contributing to the maintenance of TM integrity such as blood perfusion improvement, being nitric oxide able to increase vessel diameter and perfusion rate.

### Mitochondria

8 out of the 67 (12%) SLT-modulated genes were involved in mitochondrial functions. 7 of these genes, involved in ion transport and lipid peroxidation, were downregulated. This trend indicates a silencing of mitochondrial activities producing intracellular reactive oxygen species. However this situation does not result in energy shortage. In fact a gene (ATP5J) involved in ATP synthesis was upregulated.

### Energy production

Furthermore, among **6** genes involved in energy production (9% of 67), 3 genes, involved in glucose supply and anaerobic glycolysis, were upregulated. In parallel, 3 of these genes, involved in ATP consumption, were downregulated. These finding indicate that TM cells as a consequence of SLT exposure decrease energy consumption and increase energy production by anaerobic mechanisms. This balance results in the reduction of endogenous production of reactive oxygen species.

### Oxidative stress

8 out of 67 genes (10%) affected by SLT treatment are involved in response to oxidative stress and production of antioxidant activities. Upregulated antioxidant activities include glutathione, cyestine rich proteins, bilirubin metabolites, mitochondrial cytochrome. Two genes involved in removal of retinol derivatives were downregulated, suggesting that retinol metabolites too contribute to the increasing of the antioxidant status.

The role of reactive oxygen species in the pathogenicity of glaucoma is supported by increasing evidences [31]. Indeed, oxidative damage to the DNA of TM cells has been proved to be significantly higher in affected patients than in age-matched control subjects [32]. Oxidative damage and AH antioxidant defects mainly targets TM, which the most sensitive tissue of the anterior chamber to oxidative damage [33].

### DNA

Moreover an increased trend to DNA repair was observed. In fact among the 5 (7% of 67) genes involved in DNA repair and cell cycle, 2 involved in base repair and 1 inhibiting cell cycle were upregulated, while 3 inducing cell cycle progression were downregulated. This figure indicates a trend to cell cycle rate decrease paralleled by increased DNA repair.

Luna et al. [34] demonstrated that chronic oxidative stress may lead to increased synthesis and deposition of extracellular matrix in the TM and contribute to the elevation of intra-ocular pressure in glaucoma. Thus the trend toward oxidative damage reduction herein reported could act in synergism with the above underlined mechanisms of manteinance of the homeostasis between cells and extracellular matrix.

### Inflammation

All the 7 genes (10% out of 67) involved in inflammation and recruitment of inflammatory cells were downregulated after SLT treatment. This situation results in an immunosuppressive effect bearing relevance because inflammation has been proposed as a possible pathogenic step of POAG. These findings are in line with the ability of light to induce immunosuppression by modulating gene expression in exposed tissues [35]. Alvarado et al. [36] reported an increased expression of IL 8 in cultured TM cells treated with SLT. Unfortunately this data is not assessable in our study, the sequence of its gene not being present in our array. Furthermore Alvarado et al. recently demonstrated a monocyte recruitment in TM following SLT treatment [6] confirming a involvement of immunologic response to SLT treatment.

### Glutamate metabolism

3 out of the 67 genes modulated by SLT (4%) are involved in glutamate metabolism. This confirm a possible interaction between glutamate metabolism and the IOP in the optic nerve head where, in rabbits, the elevation of the IOP causes an increase in the glutamate levels [37]. Indeed, acute and transient intraocular hypertension induces neurodegenerative changes and the accompanying glial activation in the visual pathway. Brain changes may occur in parallel with the Retinal Ganglion Cells loss. Reactive glial cells in the brain may participate in the clearance of aberrantly released glutamate [38].

### TM gene expression

The expression of 9 genes (13%) characterizing neural tissue, as inferred from Swissprotein annotation, was well detectable expressed in TM cells and modulated by SLT treatment. These genes are identified by ‘*’ in Table S1. This finding indicate that similarities at postgenomic levels exist between TM and other neural tissues recognized as POAG targets such as optic nerve head and geniculate ganglion. TM cells have a neuro-ectodermic origin, expressing, at least in part, a neural-like phenotype [39] TM cells derive from mesenchymal cells of the neural crest [40]. This may explains why the SLT increases also the genes expression of typical neural tissues, and further confirm the similarity between TM and neural tissue previously reported comparatively analyzing proteome in TM and optic nerve head [41], [42], [43]. Furthermore, neural proteins have been specifically detected in the aqueous humour of glaucomatous patients as resulting both from TM and optic nerve head damage and these molecules may be involved in the normal formation and function of these tissues [44].

The similarity between TM and neural tissue is important for glaucoma pathogenesis because they can share common pathogenic mechanisms. The herein reported results provide evidence that glutamate cytotoxic effects representing a major pathogenic element for nerve tissues targeted by glaucoma, are also important for TM homeostasis and may be modulated in TM by SLT treatment. A trend to decreasing TM cell to the adverse glutamate effects was detected. In fact, SLT decreased the expression of gene encoding for the NMDA glutamate receptors, which mediate glutamate induced cytotoxicity. In parallel, two genes encoding for enzymes involved in glutamate catabolism were upregulated.

On the whole SLT induces in TM cell motility, increased intercellular connections, decreased extracellular matrix production, protein repair, DNA repair, membrane repair, decreased reactive oxygen species production, decreased glutamate toxicity, increased antioxidant activities, and decreased inflammation. Nevertheless it has to be taken into account that the analysis of gene expression can give a broad quantity of data but not all of them will be actually effective in cell. As a matter of that Alvarado et al. [36] pointed out that only a small portion of the differentially expressed genes are actually involved in the SLT mediated effects. Further analyses of the SLT induced modulation of the mirnoma, in progress in our laboratory, will contribute to give a more detailed picture of the mechanisms subtending the SLT efficacy and will improve the understanding of the pathogenic events of POAG. Even if the mechanism underlying contraction or relaxation of TM cells *in vivo* is not clear, it is thought that a high IOP, can extend the trabecular beams, obtaining an increased exposure of endothelial cells in AH by increasing the surface of filtration [44] and a cytokines increase into the AC that then will increase flow across the SC [36]. If the IOP falls below the venous pressure, the surface of filtration decreases and the trabecular beams become flaccid, increasing the residence to the outflow of SC [36]. Extracellular matrix proteins contribute to the homeostatic modification of aqueous humor outflow resistance and are known to be upregulated or downregulated in response to mechanical stretching [45], [46]. Our results indicate that SLT activate at least one gene encoding for a metalloprotease involved in extracellular matrix homeostasis (ADAMTS2). Other cellular changes may initially help to repair mechanical damage, and could eventually increase tissue rigidity and compromise the ability of the TM to maintain normal levels of outflow resistance [47]. TM cell adhesions, are not static structures but rather are dynamic – they come and go, vary in tightness, location, adhesivity – depending on what is going on in the environment of the cell [48]. SLT change this TM property, changing the proteins production not only structural that govern the TM functionality, in particular the assembly and disassembly of the junctions and the interactions of the cytokines that influencing the HA outflow between the TM and SC endothelium. Thus, the tri-dimensional architecture of human TM considerably increases the filtration surface, whose degeneration, resulting in the decay of HTM cellularity, causes IOP increase and triggers glaucoma pathogenesis [4]. According with Borras [41] model, the cells of the outflow pathway will initially respond to an IOP insult by triggering a defense mechanism through the activation of NF-kB. As the injury persists, as it is the case in a chronic disease, the increased activation of NF-kB will subsequently be followed by further activation of inflammatory cytokines that would then contribute to the cell damage observed in glaucoma. Particularly, chronic oxidative stress leads to the endogenous production of ROS by the mitochondria in TM cells, which in turn induces a sustained stress response characterized by activation of NF-κB and expression of inflammatory markers [49]. ROS present in AC can cause an error of replication of mtDNA determining a significant deletion of the mithocondria genome. The genome shorter replicates itself faster inducing the creation of mitochondria malfunctioning or inactive that causes an energy deficit and atrophy [50], farther mitochondria are key regulators of apoptosis [51]. The SLT lead the expression of distinct sets of genes that stimulates an improvement of TM perfomances, activating, as we have seen, genes concern the motility and the integrity of this tissue and trying to optimise the functioning of mitochondria in contrast to free radicals.

For instance, Bone morphogenetic proteins that controls multiple functions in a variety of cells are present in human optic nerve head (ONH) tissues, isolated ONH astrocytes, and Lamina Cribrosa cells [52], and can alter TGF-beta2 signaling in the TM and leads to increased ECM deposition and elevated IOP [43]. Our study confirm that glaucoma is associated with increased expression of genes that mediate axonal outgrowth, immune response, cell motility, neuroprotection, and ECM remodeling, and as glaucoma progresses, retinal ganglion cell axons may make a regenerative attempt to restore lost nerve cell contact [53]. Interestingly, some of the same genes have been reported to have an upregulated expression in the TM and lamina cribrosa cells treated with TGF, dexamethasone, mechanical stretch, or increased ocular pressure [22], [41], [42], [43], [45]. Furthermore, neural proteins have been specifically detected in the aqueous humour of glaucomatous patients as resulting both from TM and optic nerve head lamina cribrosa [39] in fact an increase in IOP resulted in morphological changes in the astrocytes [54], in a disturbances in axonal transport [55] and lead to a cytoskeletal damage in the prelaminar, lamina cribrosa, and postlaminar regions of the optic nerve, after 6 hours of raised IOP [56]. Under this light we can observe the gene expression correlated with glutamate metabolism. We know there is a way from AC and lamina cribrosa through the iris and the choroidal vessels posteriorly, [57], that may be related to glutamate receptor subunits regulating calcium fluxes, the specific pattern of neuronal vulnerability [58]. It is possible that Ionotropic glutamate receptor. L-glutamate acts as an excitatory neurotransmitter at many synapses in the central nervous system, bringing signals to the optic nerve head and leaving the vitreous relatively inert. Therefore the TM is to be considered as a organ that governs the flow from AC to SC whose functions are complex, and not unrelated to those of ciliary body whose physiopathologic meaning is now compared to a multifunctional neuroendocrine gland [59]. Anyway, it is to remember that both Aqueous Humour and plasma homocysteine levels were significantly increased in POAG, and this is a neurotoxin that induces apoptotic retinal ganglion cell death via stimulation of NMDA receptor, hence increased Hcy concentrations in AH and plasma might contribute to the optic nerve damage in POAG [60].

In conclusion SLT significantly increased the tonographic outflow facility and decreased IOP in patients with primary open angle glaucoma and ocular hypertension [61], thanks to trabecular gene-activations which involves a huge number of genes that affect mainly its metabolic functions and its micro environment. These protective effects occur without the induction of damage-related phenotypic alterations in treated TM specimens, as documented in Fig. 1.

For their absolute interest are those related to stress oxidative which confirm its pathogenetic importance [62], and those related to mitochondrial that confirm their primary role in TM function [63] and in the entire homeostasis of AC. The similarity between TM and neural tissue is important for glaucoma pathogenesis because they can share common pathogenic mechanisms. Indeed, herein reported results provide evidence that glutamate cytotoxic effects representing a major pathogenic element for nerve tissues targeted by glaucoma, are also important for TM homeostasis and may be modulated in TM by SLT treatment. Recently, our group has published a paper where it shows how the proteomic composition of aqueous humor reflects events of glaucoma pathogenesis [64]. In light of this presented results provide evidence that TM is a complex tissue possessing a great variety of function pivotal for the active regulation of aqueous humour outflow from the anterior chamber. SLT is able to modulate these function at postgenomic molecular level without inducing damage either at molecular of phenotypic level.

## Materials and Methods

### TM cell culture

Primary human TM cell line [HTM, ScienCell, San Diego, California: cat. n. 6590, see http://www.sciencellonline.com for details] were cultured in fibroblast medium (FM, ScienCell), 2% fetal bovine serum (FBS, ScienCell), fibroblast growth supplement (FGS, 1%, ScienCell) and penicillin/streptomycin solution (P/S, 1%, ScienCell) using poly-L-lysine-coated flasks having a 6 mm diameter. Cell were growth to semi confluence accounting for approximately 1×10^5^ cell per well. When reaching semi-confluence cells underwent SLT treatment.

### Selective laser trabeculoplasty treatment

TM cells underwent SLT under similar conditions usually adopted for human subjects using a SLT laser system (Laserex Tango Ellex Medical Pty Ltd – Australia). Specific treatment conditions were Q-swithched frequency doubled with Nd:YAG laser at 532 nm.

Wavelength, energy settings at 0.5 mJ single pulse, pulse duration 3 nsec, and treatment spot size of 400 micron.

After treatment cells were immediately restored in incubator and then collected by scraping after 30 min (T0.5), 2 hours (T2), and 6 hours (T6). Control cells stored in incubator in absence of SLT treatment were used as reference samples.

Scanning electron microscopic examination (Siemens Elmiskop 101; Siemens AG, Berlin, Germany) were performed on 2 Trabecular meshwork samples after SLT at 4^th^ and 6^th^ hour from treatment (Figure 1). Immediately after laser light exposition, all samples were immersed in cold antioxidant chondroitin sulfate/dextran storage medium (Optisol, Chiron Vision, Irvine, California), coded blindly, and frozen at −80°C. Trabecular meshwork samples were collected from two corneal donors in collaboration with the Melvin Jones Eye Bank of Genoa, Italy.

### Gene expression analysis by cDNA microarray

The expression of 18,401 human genes was tested in SLT-treated cells by cDNA microarray. Custom microarray as made available from the Microarray Department-University of Amsterdam, were used. The whole list of spotted genes is available at the website http://www.micro-array.nl/libraries.html. Because of the poor amount of available cell extracted RNA underwent reverse transcription and pre-plateau amplification as previously reported [65].

All data is MIAME compliant and that the raw data has been deposited in a MIAME compliant database (e.g., ArrayExpress, GEO), as detailed on the MGED Society website http://www.mged.org/Workgroups/MIAME/miame.html

Purified RNA underwent reverse transcription and amplification using quantitative real-time PCR (qPCR) prior to probe synthesis for array hybridization. A particular retrotranscription protocol (SuperSMART, Clontech, Palo Alto, CA, USA) aimed at the introduction of a target sequence at 3′ and 5′ of the cDNA library was applied as follows: RNA was incubated with a mix of a target sequence-oligo(dT)-linked primer and a target sequence-oligo(dG)-linked at 72°C for 5 minutes then a master-mix solution was added containing reverse transcriptase and dNTPs mix. Samples were then incubated at 42°C for 90 minutes. Reaction was terminated by adding ethylenediaminetetraacetic acid, mixture diluted in phosphate buffer and synthesized cDNA purified by column chromatography using a commercially available purification kit (QIAquick PCR purification kit, Qiagen, Chatsworth, CA, USA). A PCR mix, including the specific primer for the target sequences and the fluorescent tracers SYBR GREEN, was added to the purified cDNA. The PCR reaction was performed at 95°C ×1 minute, 50 amplification cycles at 95°C ×15 s, 65°C ×30 s and 68°C ×3 minutes. The amplification curve was observed in real time by recording fluorescence and the pre-plateau amplification cycle was identified for each sample. A second set of sample was amplified to the identified pre-plateau cycle using the same conditions without the fluorescent tracer. This procedure was applied to all samples in order to standardize the amplification process thus allowing reliable comparisons of microarray data among different samples. PCR reactions were performed in a thermocycler equipped with microvial rotating support (Rotorgene, Corbett Research, Mortlake, Australia).

2 ug of amplified cDNA, as purified by column chromatography, were converted in aminomodified probes performing a three cycle PCR using a PCR mix containing N-18 random primers, aminomodified dNTPs and Taq polymerase. The reaction was performed at 95°C ×5minutes, 94°C ×1 minute, 25°C ×90 s, 50°C ×10 minutes and 68°C ×5 minutes. Synthesized amino-modified oligo-nucleotides were purified by column chromatography and alcohol precipitation then labelled with fluorescent tracers Cy3 or Cy5 by incubation at room temperature in the dark for 90 minutes. Fluorescent oligonucleotides were precipitated by cold ethanol and sodium acetate, and then purified by column chromatography. The efficacy of the procedure was checked by spectrophotometric analysis measuring abosorbance at 550 (Cy3) and 650 (Cy5). Standardized amounts of labelled probes were hybridized on glass cDNA microarrays.

Probes were lyophilized, diluted in 4 ul of EDTA 10 mM and incubated at 95°C for 10 min. Hybridization solution (18 ul) was added and labelled probe mixed together to a final volume of 44 ul. Mixture was then transferred onto microarrays which were then covered with a coverslip and hybridized overnight at 50°C in a Hybridization Cassette (Life Technologies, Carlsbad, CA, USA). After 16 hours the microarrays were washed twice in a low stringency wash buffer (2X SSC, 0.5% SDS) and twice in a high stringency wash buffer (0.5X SSC, 0,5% SDS). Microarray were dried in centrifuge and signal acquired in a laser scanner (ScanArray, PerkinElmer, Waltham, MA,USA).

Data analysis was performed subtracting for each microarray the local spot background from raw spot intensity, log transformation, normalization per chip and per array (GeneSpring® software version 7.2, Agilent Technologies, Santa Clara, CA). Each gene was spotted in quadruplicate on the used microarray. Accordingly, results represent the mean among 4 data. Data generated for each mRNA were compared among the various experimental groups by volcano-plot analysis taking into account as thresholds of two-fold variation and statistical significance P<0.05 as evaluated by ANOVA after Bonferroni multiple testing correction. Global mRNA expression profiles were compared by hierarchical cluster analysis (HC) and bidimensional principal component analysis of variance (PCA).

Gene function was inferred from annotation reported in gene databank (Weizmann Institute, www.genecards.org) and Swissprotein database (http://expasy.org/sprot/).

“Biological function of genes whose expression was modulate by SLT was inferred from data available on Swissprot (http://expasy.org/sprot/) and Weizmann Institute (http://www.genecards.org/) databases. Predictor genes were identified by statistical support vector machine analysis, which is a supervised learning methods that predicts, for each given input, which of possible classes the input is a member of identifying the most relevant genes affecting this classification”.

## Supporting Information

Table S1 **Gene whose expression in TM cells was modulated by SLT treatment (control vs. SLT).** 89 genes.
(DOC)
