# Supplementary material for: Trabecular Meshwork Gene Expression after Selective Laser Trabeculoplasty
Source: PLoS One. 2011 Jul 1;6(7):e20110. doi: 10.1371/journal.pone.0020110 (PMC3128580; doi:10.1371/journal.pone.0020110)
Supplement: Table S1 — Gene whose expression in TM cells was modulated by SLT treatment (control vs. SLT). 89 genes. (DOC) [file pone.0020110.s001.doc]

**Table 1**. Gene whose expression in TM cells was modulated by SLT treatment (control vs SLT). 89 genes.

| **Activated protective mechanism** | **Gene name** | **Gene code** | | **SLT induced fold-variation trend**  **signal intensity FU°**  **(SLT/Control ratio)** | | | | **Ontology** | **Function** |
| --- | --- | --- | --- | --- | --- | --- | --- | --- | --- |
|  |  | **GeneBank** | **UniGene** | **T0**  **hours** | **T0.5 hours** | **T2 hours** | **T6 hours** |  |  |
| ***TM MOTILITY*** |  |  |  |  |  |  |  |  |  |
| Increased TM palsticity | Beta-actin | D28354 | [Hs.520640](http://www.ncbi.nlm.nih.gov/UniGene/clust.cgi?ORG=Hs&CID=520640) | 0.7 | 0.8  (=1.2) | 14.9  (↑ 22.2) | 11.9  (↑17.8) | Cell motility | Cell adhesion and motility |
| Maintenance of TM integrity | LIM and SH3 protein 1 (LASP1) | NM_006148 | [Hs.725954](http://www.ncbi.nlm.nih.gov/UniGene/clust.cgi?ORG=Hs&CID=725954) | 1.7 | 3.4  (↑2.0) | 83.6  (↑49.2) | 79.0  (↑46.5) | Tissue plasticity | Regulation of dynamic actin-based, cytoskeletal activities. |
| Decreased resistance to aqueous humour outflow | Myosin, light polypeptide kinase (MYLK)* | NM_005965 | [Hs.477375](http://www.ncbi.nlm.nih.gov/UniGene/clust.cgi?ORG=Hs&CID=477375) | 1.1 | 4.9  (↑4.6) | 33.6  (↑31.1) | 37.8  (↑35.0) | TM motility | Calcium/calmodulin-dependent enzyme implicated in smooth muscle contraction via phosphorylation of myosin light chains . Also regulates actin-myosin interaction through a non-kinase activity. Implicated in the regulation of endothelial as well as vascular permeability. In the nervous system it has been shown to control the growth initiation of astrocytic. |
| Decreased resistance to aqueous humour outflow | Microfibril-associated glycoprotein-2 (MAGP2) | NM_003480 | [Hs.512842](http://www.ncbi.nlm.nih.gov/UniGene/clust.cgi?ORG=Hs&CID=512842) | 1.5 | 9.0  (↑5.9) | 57.8  (↑37.6) | 54.1  (↑35.2) | TM motility | Component of the elastin-associated microfibrils. |
| Increased TM plasticity | Calponin 1, basic, smooth muscle (CNN1) | NM_001299 | [Hs.465929](http://www.ncbi.nlm.nih.gov/UniGene/clust.cgi?ORG=Hs&CID=465929) | 1.0 | 0.8  (=1.2) | 71.0  (↑72.3) | 54.3  (↑55.3) | Cell motility | Thin filament-associated protein that is implicated in the regulation and modulation of smooth muscle contraction. It is capable of binding to actin, calmodulin, troponin C and tropomyosin. |
| Increased TM plasticity | Tropomodulin 2 (neuronal) (TMOD2) | NM_014548 | [Hs.513734](http://www.ncbi.nlm.nih.gov/UniGene/clust.cgi?ORG=Hs&CID=513734) | 1.3 | 2.7  (↑2.1) | 36.1  (↑28.5) | 26.8  (↑21.1) | Cell motility | Regulates elongation and depolymerization of the actin filaments at the pointed end. The Tmod/TM complex contributes to the formation of the short actin protofilament, which in turn defines the geometry of the membrane skeleton |
| Increased TM plasticity | Tropomyosin 1 (alpha) (TPM1) | NM_000366 | [Hs.133892](http://www.ncbi.nlm.nih.gov/UniGene/clust.cgi?ORG=Hs&CID=133892) | 0.8 | 0.9  (=1.1) | 22.6  (↑27.9) | 19.1  (↑23.6) | Cell motility | Binds to actin filaments in muscle and non-muscle cells. Plays a central role, in association with the troponin complex, in the calcium dependent regulation of muscle contraction. In non-muscle cells is implicated in stabilizing cytoskeleton actin filaments. |
|  |  |  |  |  |  |  |  |  |  |
| ***TM INTEGRITY*** |  |  |  |  |  |  |  |  |  |
| Decreased resistance to aqueous humour outflow | Heparan sulfate proteoglycan core protein | J04621 | [Hs.562227](http://www.ncbi.nlm.nih.gov/UniGene/clust.cgi?ORG=Hs&CID=562227) | 3.9 | 20.4  (↑ 5.2) | 81.2  (↑ 20.8) | 61.4  (↑ 15.7) | Tissue plasticity | Integral component of endothelia basement membranes. Responsible for the fixed negative electrostatic membrane charge providing a size- and charge-selective barrier. It serves as an attachment substrate for cells. |
| Maintenance of TM integrity | Vesicle-associated membrane protein 3 (cellubrevin) (VAMP3) | NM_004781 | [Hs.66708](http://www.ncbi.nlm.nih.gov/UniGene/clust.cgi?ORG=Hs&CID=66708) | 3.6 | 5.8  (=1.6) | 109.6  (↑ 30.4) | 97.2  (↑ 26.9) | Tissue integrity | Trafficking protein from a constitutively recycling pathway. Intercellular Cell junction and communication |
| Maintenance of TM integrity | A kinase (PRKA) anchor protein 8 (AKAP8), mRNA | NM_005858 | [Hs.726377](http://www.ncbi.nlm.nih.gov/UniGene/clust.cgi?ORG=Hs&CID=726377) | 10.9 | 4.5  (↓2.4) | 144.6  (↑13.3) | 159.6  (↑14.7) | Signal transduction  Intercellular connection | Anchoring protein that mediates the subcellular compartmentation of cAMP-dependent protein kinase (PKA type II). |
| Maintenance of TM integrity | Amine oxidase, copper containing 3 (vascular adhesion protein 1) (AOC3), mRNA | NM_003734 | [Hs.198241](http://www.ncbi.nlm.nih.gov/UniGene/clust.cgi?ORG=Hs&CID=198241) | 1.7 | 2.1  (=1.2) | 166.5  (↑95.8) | 167.0  (↑96.1) | Intercellular connection | Cell adhesion protein |
| Maintenance of TM integrity | Integrin, alpha 3 (ITGA3) | NM_002204 | Hs.265829 | 1.0 | 2.2  (↑2.2) | 9.7  (↑9.9) | 13.6  (↑13.9) | Intercellular connection | Receptor for fibronectin, laminin, collagen, epiligrin, thrombospondin. Mediates endothelial cells migration |
| Maintenance of TM integrity | Integrin, beta-like 1 (with EGF-like repeat domains) (ITGBL1) | NM_004791 | [Hs.696554](http://www.ncbi.nlm.nih.gov/UniGene/clust.cgi?ORG=Hs&CID=696554) | 0.7 | 1.0  (=1.6) | 37.7  (↑57.8) | 125.3  (↑92.5) | Intercellular connection | Receptor for fibronectin, laminin, collagen |
| Maintenance of TM integrity | Protocadherin 9 (PCDH9) | NM_020403 | [Hs.654709](http://www.ncbi.nlm.nih.gov/UniGene/clust.cgi?ORG=Hs&CID=654709) | 0.8 | 0.6  (=1.3) | 8.9  (↑11.7) | 11.6  (↑15.2) | Intercellular adhesion | Cell-adhesion protein. |
| Increased aqueous humour outflow | Connexin 31 (GJB3) | AF_052692 | [Hs.522561](http://www.ncbi.nlm.nih.gov/UniGene/clust.cgi?ORG=Hs&CID=522561) | 5.1 | 4.3  (=1.2) | 0.8  (↓6.1) | 0.3  (↓18.2) | Tissue permeability | Component of connexons, through which materials of low molecular weight diffuse from one cell to a neighboring cell. |
| Maintenance of TM integrity | UDP-glucose dehydrogenase (UGDH) | NM_003359 | [Hs.572518](http://www.ncbi.nlm.nih.gov/UniGene/clust.cgi?ORG=Hs&CID=572518) | 5.1 | 2.3  (↓ 2.2) | 1.2  (↓ 4.3) | 0.1  (↓ 51.4) | Production of extracellular matrix | Involved in the biosynthesis of glycosaminoglycans; hyaluronan, chondroitin sulfate, and heparan sulfate. |
| Decreased resistance to aqueous humour outflow | Integrin, alpha 2 (CD49B, alpha 2 subunit of VLA-2 receptor) (ITGA2) | NM_002203 | [Hs.482077](http://www.ncbi.nlm.nih.gov/UniGene/clust.cgi?ORG=Hs&CID=482077) | 7.8 | 16.3  (↑2.1) | 1.0  (↓7.5) | 0.6  (↓12.9) | Production of extracellular matrix | Receptor for laminin, collagen, collagen C-propeptides, fibronectin and E-cadherin. Responsible for adhesion of platelets and other cells to collagens, modulation of collagen and collagenase gene expression, force generation and organization of newly synthesized extracellular matrix. |
| Increased intercellular direct connections | Disintegrin-like and metalloprotease with thrombospondin type 1 motif, 2 (ADAMTS2) | NM_014244 | [Hs.23871](http://www.ncbi.nlm.nih.gov/UniGene/clust.cgi?ORG=Hs&CID=23871) | 2.8 | 15.6  (↑5.5) | 206.5  (↑73.5) | 209.3  (↑74.5) | Intercellular matrix digestion | Cleaves the propeptides of type I and II collagen prior to fibril assembly. Does not act on type III collagen. |
| Removal of damaged proteins | Cathepsin D (lysosomal aspartyl protease) (CTSD) | NM_001909 | [Hs.654447](http://www.ncbi.nlm.nih.gov/UniGene/clust.cgi?ORG=Hs&CID=654447) | 1.9 | 7.9  (↑4.2) | 135.3  (↑71.0) | 171.5  (↑90.0) | Protein repair | Acid protease active in intracellular protein breakdown |
| Removal of damaged proteins | Proteasome subunit, alpha type, 4 (PSMA4) | NM_002789 | [Hs.251531](http://www.ncbi.nlm.nih.gov/UniGene/clust.cgi?ORG=Hs&CID=251531) | 4.7 | 6.6  (=1.4) | 24.0  (↑5.1) | 102.9  (↑21.9) | Protein repair | The proteasome is a multicatalytic proteinase complex which is characterized by its ability to cleave peptides with Arg, Phe, Tyr, Leu, and Glu adjacent to the leaving group at neutral or slightly basic pH. The proteasome has an ATP-dependent proteolytic activity. |
| Removal of damaged proteins | Protein-L-isoaspartate (D-aspartate) O-methyltransferase (PCMT1) | NM_005389 | [Hs.279257](http://www.ncbi.nlm.nih.gov/UniGene/clust.cgi?ORG=Hs&CID=279257) | 2.2 | 6.0  (↑2.7) | 166.2  (↑75.2) | 206.7  (↑93.5) | Protein repair | Catalyzes the methyl esterification of L-isoaspartyl and D-aspartyl residues in peptides and proteins that result from spontaneous decomposition of normal L-aspartyl and L-asparaginyl residues. It plays a role in the repair and/or degradation of damaged proteins. |
| Removal of damaged proteins | ADP-ribosylation factor domain protein 1, 64kD (ARFD1) | NM_001656 | [Hs.792](http://www.ncbi.nlm.nih.gov/UniGene/clust.cgi?ORG=Hs&CID=792) | 2.2 | 5.3  (↑2.4) | 274.0  (↑122.2) | 290.6  (↑129.6) | Protein repair | Membrane-associated with the Golgi complex and lysosomal structures. |
| Decreased apoptosis activation | Calcium channel, voltage-dependent, alpha 1G subunit (CACNA1G) | NM_018896 | [Hs.591169](http://www.ncbi.nlm.nih.gov/UniGene/clust.cgi?ORG=Hs&CID=591169) | 52.3 | 0.6  (↓88.5) | 4.2  (↓12.5) | 0.5  (↓112.2) | Decreased calcium sensitivity | Voltage-sensitive calcium channels mediate the entry of calcium ions into excitable cells and are also involved in a variety of calcium-dependent processes, including cell death. |
| Decreased apoptosis activation | Calcium channel, voltage-dependent, L type, alpha 1B subunit (CACNA1B) | NM_000718 | [Hs.495522](http://www.ncbi.nlm.nih.gov/UniGene/clust.cgi?ORG=Hs&CID=495522) | 7.9 | 5.6  (=1.4) | 1.2  (↓6.6) | 0.5  (↓15.3) | Decreased calcium sensitivity | Voltage-sensitive calcium channels (VSCC) mediate the entry of calcium ions into excitable cells and are also involved in a variety of calcium-dependent processes, including cell death. |
| Decreased apoptosis activation | Guanylate cyclase activator 1C (GUCA1C)* | NM_005459 | [Hs.233363](http://www.ncbi.nlm.nih.gov/UniGene/clust.cgi?ORG=Hs&CID=233363) | 4.5 | 17.7  (↑3.9) | 93.3  (↑20.5) | 98.1  (↑21.6) | Decreased calcium sensitivity | Stimulates guanylyl cyclase 1 (GC1) and GC2 when free calcium ions concentration is low and inhibits guanylyl cyclases when free calcium ions concentration is elevated. This Ca2+-sensitive regulation of guanylyl cyclase (GC) is a key event in recovery of the dark state of rod photoreceptors following light exposure. |
| Decreased apoptosis activation | Potassium large conductance calcium-activated channel, subfamily M, beta member 3-like (KCNMB3L) | NM_014406 | [Hs.128342](http://www.ncbi.nlm.nih.gov/UniGene/clust.cgi?ORG=Hs&CID=128342) | 5.9 | 2.2  (↓2.7) | 0.2  (↓29.4) | 0.1  (↓59.5) | Decreased calcium sensitivity | Calcium-activated potassium channel activity |
| Maintenance of membrane fluidity | Collagen type I receptor, (CD36L1) | NM_005505 | [Hs.725979](http://www.ncbi.nlm.nih.gov/UniGene/clust.cgi?ORG=Hs&CID=725979) | 0.7 | 1.2  (=1.6) | 70.4  (↑95.4) | 80.7  (↑109.3) | Lipid uptake | Receptor for different ligands such as phospholipids, cholesterol ester, lipoproteins, phosphatidylserine and apoptotic cells. Involved in the phagocytosis of apoptotic cells. |
| Maintenance of TM integrity | Transmembrane glycoprotein (c-fms) gene, exon 1, and platelet-derived growth factor receptor (PDGF)* | M25785 | [Hs.1976](http://www.ncbi.nlm.nih.gov/UniGene/clust.cgi?ORG=Hs&CID=1976) | 3.0 | 14.2  (↑4.7) | 91.1  (↑30.5) | 106.7  (↑35.7) | Tissue repair | Potent mitogen for cells of mesenchymal origin. Binding of this growth factor to its affinity receptor elicits a variety of cellular responses. It is released by platelets upon wounding and plays an important role in stimulating adjacent cells to grow and thereby heals the wound. Expressed also in neural and other tissues. |
| Maintenance of TM integrity | Solute carrier family 12 (sodium/potassium/chloride transporters), member 2 (SLC12A2) | NM_001046 | [Hs.162585](http://www.ncbi.nlm.nih.gov/UniGene/clust.cgi?ORG=Hs&CID=162585) | 5.2 | 6.3  (=1.2) | 69.2  (↑13.3) | 65.9  (↑12.7) | Ion exchange  Cell viability | Mediates sodium and chloride reabsorption. Plays a vital role in the regulation of ionic balance and cell volume. |
| Maintenance of TM integrity  Increased TM perfusion | Solute carrier family 3 (activators of dibasic and neutral amino acid transport), member 2 (SLC3A2)* | NM_002394 | [Hs.502769](http://www.ncbi.nlm.nih.gov/UniGene/clust.cgi?ORG=Hs&CID=502769) | 1.0 | 3.2  (↑3.2) | 169.4  (↑52.3) | 199.3  (↑72.7) | Aminoacids uptake  Nitric oxide synthesis  Cell growth | Involved in sodium-independent, high-affinity transport of amino acids such as phenylalanine, tyrosine, leucine, arginine and tryptophan. Plays a role in nitric oxide synthesis via transport of L-arginine. Required for normal and neoplastic cell growth. When associated with SLC7A5, plays an important role in transporting L-leucine from the circulating blood to the retina across the inner blood-retinal barrier |
|  |  |  |  |  |  |  |  |  |  |
| ***MITOCHONDRION FUNCTION*** |  |  |  |  |  |  |  |  |  |
| Maintenance of mitochondrial integrity | Voltage-dependent anion channel 3 (VDAC3) | NM_005662 | [Hs.655340](http://www.ncbi.nlm.nih.gov/UniGene/clust.cgi?ORG=Hs&CID=655340) | 5.4 | 8.2  (= 1.5) | 0.9  (↓5.7) | 0.1  (↓ 53.9) | Mitochondrial function | Forms a channel through the mitochondrial outer membrane that allows diffusion of small hydrophilic molecules and ion transport. |
| Decreased endogenous lipid peroxdiation | Acyl-Coenzyme A dehydrogenase, C-4 to C-12 straight chain (ACADM) | NM_000016 | [Hs.445040](http://www.ncbi.nlm.nih.gov/UniGene/clust.cgi?ORG=Hs&CID=445040) | 0.6 | 0.8  (=1.2) | 1.1  (=1.8) | 0.1  (↓6.2) | Fatty acid metabolism | Specific for acyl chain lengths of 4 to 16. Located in mitochondrial matrix. Nuclear gene encoding mitochondrial protein. |
| Increased mitochondrial function | ATP synthase, H+ transporting, mitochondrial F0 complex, subunit F6 (ATP5J) | NM_001685 | [Hs.246310](http://www.ncbi.nlm.nih.gov/UniGene/clust.cgi?ORG=Hs&CID=246310) | 0.8 | 1.9  (↑2.3) | 12.0  (↑14.5) | 9.7  (↑11.8) | Energy production | Mitochondrial membrane ATP synthase. Produces ATP from ADP in the presence of a proton gradient across the membrane which is generated by electron transport complexes of the respiratory chain. |
| Maintenance of mitochondrial integrity | Carbonic anhydrase VA mitochondrial (CA5A) | NM_001739 | [Hs.177446](http://www.ncbi.nlm.nih.gov/UniGene/clust.cgi?ORG=Hs&CID=177446) | 1.9 | 1.3  (=1.5) | 1.6  (=1.2) | 0.1  (↓18.8) | Mitochondrial function | Reversible hydration of carbon dioxide. Located in mitochondrial matrix, |
| Decreased endogenous production of reactive oxygen species | Mitochondrial Fatty-acid-Coenzyme A ligase, long-chain 1 (FACL1) | NM_001995 | [Hs.406678](http://www.ncbi.nlm.nih.gov/UniGene/clust.cgi?ORG=Hs&CID=406678) | 9.1 | 24.2  (= 1.9) | 1.3  (↓6.9) | 0.5  (↓17.1) | Lipid beta oxidation | Activation of long-chain fatty acids for both synthesis of cellular lipids, and degradation via beta-oxidation. |
| Decreased endogenous lipid peroxdiation | Homo sapiens acetyl-Coenzyme A carboxylase beta (ACACB), mRNA | NM_001093 | [Hs.234898](http://www.ncbi.nlm.nih.gov/UniGene/clust.cgi?ORG=Hs&CID=234898) | 0.9 | 0.7  (=1.3) | 1.1  (=1.5) | 0.1  (↓8.8) | Fatty acid oxidation | Involved in the provision of malonyl-CoA or in the regulation of fatty acid oxidation, rather than fatty acid biosynthesis. Carries out three functions: biotin carboxyl carrier protein, biotin carboxylase and carboxyltransferase. |
| Decreased enodegenous production of reactive oxygen species | Mitochondrial Ferredoxin reductase (FDXR) | NM_004110 | [Hs.69745](http://www.ncbi.nlm.nih.gov/UniGene/clust.cgi?ORG=Hs&CID=69745) | 0.5 | 0.8  (=1.5) | 0.9  (=1.7) | 0.1  (↓5.4) | Mitochondrial function | Serves as the first electron transfer protein in all the mitochondrial P450 systems. |
| Decreased endogenous production of reactive oxygen species | Glutamic-oxaloacetic transaminase 2, mitochondrial (aspartate aminotransferase 2) (GOT2) | NM_002080 | [Hs.599470](http://www.ncbi.nlm.nih.gov/UniGene/clust.cgi?ORG=Hs&CID=599470) | 1.6 | 2.2  (=1.4) | 1.3  (=1.2) | 0.1  (↓16.4) | Mitochondrial lipid transmembrane transport | Plays a key role in amino acid metabolism. Important for metabolite exchange between mitochondria and cytosol. Facilitates cellular uptake of long-chain free fatty acids. |
|  |  |  |  |  |  |  |  |  |  |
| ***ENERGY PRODUCTION*** |  |  |  |  |  |  |  |  |  |
| ATP production without endogenous formation of reactive oxygen species | Lactate dehydrogenase B (LDHB) | NM_002300 | [Hs.446149](http://www.ncbi.nlm.nih.gov/UniGene/clust.cgi?ORG=Hs&CID=446149) | 4.4 | 19.3  (↑4.4) | 277.5  (↑62.7) | 298.0  (↑67.3) | Anaerobic glycolysis | Catalyzes the interconversion of [pyruvate](http://en.wikipedia.org/wiki/Pyruvate) and [lactate](http://en.wikipedia.org/wiki/Lactic_acid) with concomitant interconversion of NADH and [NAD^+^](http://en.wikipedia.org/wiki/Nicotinamide_adenine_dinucleotide). It converts pyruvate, the final product of [glycolysis](http://en.wikipedia.org/wiki/Glycolysis) to lactate when oxygen is absent or in short supply. |
| ATP production without endogenous formation of reactive oxygen species | Malate dehydrogenase 1, NAD (soluble) (MDH1) | NM_005917 | [Hs.526521](http://www.ncbi.nlm.nih.gov/UniGene/clust.cgi?ORG=Hs&CID=526521) | 6.9 | 6.1  (=1.1) | 89.4  (↑13.0) | 85.2  (↑12.4) | Anaerobic glycolisis | Oxidoreductase |
| Increased intracellular glucose availability | Glucose-6-phosphatase, catalytic (G6PC) | NM_000151 | [Hs.212293](http://www.ncbi.nlm.nih.gov/UniGene/clust.cgi?ORG=Hs&CID=212293) | 1.0 | 1.7  (=1.7) | 20.8  (↑21.4) | 17.8  (↑18.3) | Glucose supplying | Hydrolyzes glucose-6-phosphate to glucose in the endoplasmic reticulum. Forms with the glucose-6-phosphate transporter (SLC37A4/G6PT) the complex responsible for glucose production through glycogenolysis and gluconeogenesis. |
| Decreased ATP consumption | ATPase, Na+/K+ transporting, alpha 1 polypeptide (ATP1A1) | NM_000701 | [Hs.371889](http://www.ncbi.nlm.nih.gov/UniGene/clust.cgi?ORG=Hs&CID=371889) | 1.0 | 1.4  (=1.4) | 1.3  (=1.3) | 0.1  (↓9.5) | Transmembrane ion transport | This is the catalytic component of the active enzyme, which catalyzes the hydrolysis of ATP coupled with the exchange of sodium and potassium ions across the plasma membrane. This action creates the electrochemical gradient of sodium and potassium ions, providing the energy for active transport of various nutrients. |
| Decreased ATP consumption | ATPase, Na+/K+ transporting, beta 2 polypeptide (ATP1B2) | NM_001678 | [Hs.643540](http://www.ncbi.nlm.nih.gov/UniGene/clust.cgi?ORG=Hs&CID=643540) | 1.0 | 0.9  (=1.1) | 1.3  (=1.3) | 0.1  (↓10.4) | Transmembrane ion transport | This is the non-catalytic component of the active enzyme, which catalyzes the hydrolysis of ATP coupled with the exchange of Na+ and K+ ions across the plasma membrane. |
| Decreased ATP consumption | ATP-binding cassette, sub-family B (MDR/TAP), member 8 (ABCB8) | NM_007188 | [Hs.647118](http://www.ncbi.nlm.nih.gov/UniGene/clust.cgi?ORG=Hs&CID=647118) | 0.9 | 1.0  (=1.1) | 0.3  (=1.3) | 0.1  (↓10.4) | Transmembrane transport | ATPase activity,coupled to transmembrane movement of substances |
|  |  |  |  |  |  |  |  |  |  |
| ***OXIDATIVE STRESS*** |  |  |  |  |  |  |  |  |  |
| Increased antioxidant defense | Biliverdin reductase B (flavin reductase (NADPH)) (BLVRB) | NM_000713 | [Hs.515785](http://www.ncbi.nlm.nih.gov/UniGene/clust.cgi?ORG=Hs&CID=515785) | 6.8 | 9.2  (=1.1) | 0.6  (=1.3) | 0.3  (↑10.4) | Antioxidant | Broad specificity oxidoreductase that catalyzes the NADPH-dependent reduction of a variety of flavins, such as riboflavin, FAD or FMN, biliverdins, methemoglobin and pyrroloquinoline quinone. Reduce the complexed Fe3+ iron to Fe2+ in the presence of FMN and NADPH |
| Increased antioxidant defense | Carbonyl reductase 1 (CBR1) | NM_001757 | [Hs.606200](http://www.ncbi.nlm.nih.gov/UniGene/clust.cgi?ORG=Hs&CID=606200) | 1.2 | 1.4  (= 1.2) | 35.0  (↑29.8) | 28.8  (↑24.5) | Glutathione reduction | NADPH-dependent reductase with broad substrate specificity. Catalyzes the reduction of a wide variety of carbonyl compounds including quinones, prostaglandins.  Convert prostaglandin E2 to prostaglandin F2-alpha. Bind glutathione. High affinity for glutathione-conjugated substrates. Catalyzes the reduction of S-nitrosoglutathione |
| Protection against hypoxic injury | Cystathionine-beta-synthase (CBS) | NM_000071 | [Hs.533013](http://www.ncbi.nlm.nih.gov/UniGene/clust.cgi?ORG=Hs&CID=533013) | 2..0 | 3.2  (=1.6) | 174.6  (↑87.7) | 186.4  (↑93.6) | Cell protection  Antioxidant | Pyridoxal phosphate- heme-containing enzyme. Important regulator of hydrogen sulfide, especially in the brain, utilizing cysteine instead of serine to catalyze the formation of hydrogen sulfide having cytoprotective effects and protecting cells against hypoxic injury. |
| Increased antioxidant defense | Glutathione S-transferase (subunit 13 homolog) | NM_015917 | [Hs.390667](http://www.ncbi.nlm.nih.gov/UniGene/clust.cgi?ORG=Hs&CID=390667) | 10.9 | 18.7  (=1.7) | 166.7  (↑15.3) | 158.0  (↑14.5) | Antioxidant | Protein disulfide oxidoreductase activity |
| Increased antioxidant defense | Cytochrome b5 | AB009282 | [Hs.461131](http://www.ncbi.nlm.nih.gov/UniGene/clust.cgi?ORG=Hs&CID=461131) | 10.9 | 40.7  (↑3.7) | 360.3  (↑32.9) | 387.7  (↑35.4) | Antioxidant | NADH-cytochrome b5 reductase involved in endolasmic reticulum stress response pathway. Plays a critical role in protecting against oxidant stress, possibly by protecting the cell from excess buildup of reactive oxygen species (ROS). Located in mitochondrial outer membrane. |
| Increased antioxidant defense | Cytochrome P450 retinoid metabolizing protein (P450RAI-2) | NM_019885 | [Hs.91546](http://www.ncbi.nlm.nih.gov/UniGene/clust.cgi?ORG=Hs&CID=91546) | 0.8 | 0.6  (=1.4) | 0.6  (= 1.4) | 0.1  (↓7.8) | Retinoic acid catabolism | Plays a key role in retinoic acid metabolism. Involved in the specific inactivation of retinoic acid. |
| Increased antioxidant defense | Cytochrome P450, subfamily XXVIA, polypeptide 1 (CYP26A1) | NM_000783 | [Hs.150595](http://www.ncbi.nlm.nih.gov/UniGene/clust.cgi?ORG=Hs&CID=150595) | 1.4 | 1.1  (=1.3) | 0.8  (=1.8) | 0.1  (↓13.7) | Retinoic acid catabolism | Plays a key role in retinoic acid metabolism. Acts on retinoids, including all-trans-retinoic acid (RA) and its stereoisomer 9-cis-RA. Involved in the specific inactivation of retinoic acid. |
| Maintenance of in tracellular pH | Na+/H+ exchanger isoform 2 (NHE2) | AF073299 | [Hs.250083](http://www.ncbi.nlm.nih.gov/UniGene/clust.cgi?ORG=Hs&CID=250083) | 4.0 | 15.9  (↑4.0) | 58.9  (↑14.8) | 50.5  (↑20.2) | Stress response | Involved in pH regulation to eliminate acids generated by active metabolism or to counter adverse environmental conditions. Major proton extruding system driven by the inward sodium ion chemical gradient. |
|  |  |  |  |  |  |  |  |  |  |
| ***DNA REPAIR and CELL CYCLE*** |  |  |  |  |  |  |  |  |  |
| Repair of oxidatively damaged nucleotides | ADP-ribosyltransferase (NAD+; poly (ADP-ribose) polymerase)-like 3 (ADPRTL3) | NM_005485 | [Hs.271742](http://www.ncbi.nlm.nih.gov/UniGene/clust.cgi?ORG=Hs&CID=271742) | 2.1 | 3.1  (=1.5) | 30.2  (↑14.7) | 30.0  (↑14.3) | DNA repair | Involved in the base excision repair (BER) pathway, by catalyzing the poly(ADP-ribosyl)ation of a limited number of acceptor proteins involved in chromatin architecture and in DNA metabolism. This modification follows DNA damages and appears as an obligatory step in a detection/signaling pathway leading to the reparation of DNA strand breaks. |
| Increased DNA repair | Polymerase (DNA directed) iota (POLI) | NM_007195 | [Hs.438533](http://www.ncbi.nlm.nih.gov/UniGene/clust.cgi?ORG=Hs&CID=438533) | 2.4 | 4.8  (=1.9) | 66.9  (↑26.7) | 58.4  (↑23.3) | DNA repair | Error-prone DNA polymerase specifically involved in DNA repair. Plays an important role in translesion synthesis, where the normal high-fidelity DNA polymerases cannot proceed and DNA synthesis stalls. Inserts the correct base with high-fidelity opposite an adenosine template. Exhibits low fidelity and efficiency opposite a thymidine template, where it will preferentially insert guanosine. |
| Decreased cell cycle rate allowing increased DNA repair | Cdk inhibitor p21 binding protein (TOK-1) | NM_016567 | [Hs.381189](http://www.ncbi.nlm.nih.gov/UniGene/clust.cgi?ORG=Hs&CID=381189) | 4.8 | 25.3  (↑5.2) | 293.2  (↑60.8) | 374.7  (↑77.7) | DNA repair | Promote cell cycle arrest by enhancing the inhibition of CDK2 activity by CDKN1A. Required for repair of DNA damage by homologous recombination. |
| Decreased cell cycle rate allowing increased DNA repair | Cyclin-dependent kinase 7 (CDK7) | NM_001799 | [Hs.184298](http://www.ncbi.nlm.nih.gov/UniGene/clust.cgi?ORG=Hs&CID=184298) | 6.6 | 4.4  (=1.5) | 0.6  (↓11.0) | 0.5  (↓12.7) | Cell cycle progression | Cyclin-dependent kinases (CDKs) are activated by the binding to a cyclin and mediate the progression through the cell cycle. |
| Decreased cell cycle rate allowing increased DNA repair | Protein regulator of cytokinesis 1 (PRC1) | NM_003981 | [Hs.366401](http://www.ncbi.nlm.nih.gov/UniGene/clust.cgi?ORG=Hs&CID=366401) | 1.4 | 3.5  (= 1.9) | 2.0  (=1.5) | 0.1  (↓13.9) | Cell cycle progression | Plays a role in the construction of spindle microtubules during the metaphase to anaphase transition, an essential step for the formation of an organized central spindle midzone and midbody and for successful cytokinesis. May function as an in vivo cyclin-CDK substrate. |
|  |  |  |  |  |  |  |  |  |  |
| ***GLUTAMATE METABOLISM*** |  |  |  |  |  |  |  |  |  |
| Decreased endogenous cellular toxicity | NMDA receptor glutamate-binding chain* | U44954 | [Hs.594634](http://www.ncbi.nlm.nih.gov/UniGene/clust.cgi?ORG=Hs&CID=594634) | 4.5 | 2.1  (↓ 2.2) | 1.5  (↓ 3.1) | 0.1  (↓ 44.7) | Glutamate receptor | Ionotropic glutamate receptor. L-glutamate acts as an excitatory neurotransmitter at many synapses in the central nervous system. Binding of the excitatory neurotransmitter L-glutamate induces a conformation change, leading to the opening of the cation channel, and thereby converts the chemical signal to an electrical impulse. Glutamate mediates cell toxicity binding to NMDA receptor. |
| Decreased endogenous cellular toxicity | Glutamate-cysteine ligase, modifier subunit (GCLM)* | NM_002061 | [Hs.315562](http://www.ncbi.nlm.nih.gov/UniGene/clust.cgi?ORG=Hs&CID=315562) | 1.0 | 2.4  (↑2.4) | 24.7  (↑25.7) | 19.5  (↑20.3) | Glutamate catabolism | Glutamate-cysteine ligase catalytic subunit |
| Decreased endogenous cellular toxicity | Glial high affinity glutamate transporter, member 3 (SLC1A3)* | NM_004172 | [Hs.481918](http://www.ncbi.nlm.nih.gov/UniGene/clust.cgi?ORG=Hs&CID=481918) | 0.7 | 1.1  (=1.6) | 11.0  (↑15.2) | 11.3  (↑15.7) | Glutamate catabolism | Transports L-glutamate and also L- and D-aspartate. Essential for terminating the postsynaptic action of glutamate by rapidly removing released glutamate. Acts as a symport by cotransporting sodium. Nuclear gene encoding mitochondrial protein. |
|  |  |  |  |  |  |  |  |  |  |
| ***INFLAMMATION*** |  |  |  |  |  |  |  |  |  |
| Immunosuppression | H.sapiens mRNA for CD22 protein | Y10210 | [Hs.579691](http://www.ncbi.nlm.nih.gov/UniGene/clust.cgi?ORG=Hs&CID=579691) | 2.7 | 13.6  (↑5.0) | 1.1  (↓2.4) | 0.1  (↓27.4) | Inflammation | Mediates B-cell B-cell interactions. Involved in regulation of B-cell antigen receptor signaling. |
| Immunosuppression | C-type (calcium dependent, carbohydrate-recognition domain) lectin, superfamily member 9 (CLECSF9) | NM_014358 | [Hs.236516](http://www.ncbi.nlm.nih.gov/UniGene/clust.cgi?ORG=Hs&CID=236516) | 0.7 | 1.0  (=1.4) | 1.2  (=1.6) | 0.1  (↓7.4) | Inflammation | Plays a role in the response to inflammatory stimuli. |
| Immunosuppression | C-type (calcium dependent, carbohydrate-recognition domain) lectin, superfamily member 6 (CLECSF6) | NM_016184 | [Hs.504657](http://www.ncbi.nlm.nih.gov/UniGene/clust.cgi?ORG=Hs&CID=504657) | 6.5 | 11.8  (=1.8) | 0.7  (↓9.9) | 0.6  (↓11.6) | Inflammation | Involved in regulating immune reactivity and dendritic cell activation. |
| Immunosuppression | Endothelial differentiation, G-protein-coupled receptor 6 (EDG6) | NM_003775 | [Hs.662006](http://www.ncbi.nlm.nih.gov/UniGene/clust.cgi?ORG=Hs&CID=662006) | 2.3 | 2.8  (=1.2) | 1.7  (=1.4) | 0.1  (↓23.4) | Lymphocyte recruitment | Receptor for the lysosphingolipid sphingosine 1-phosphate, a bioactive lysophospholipid that elicits diverse physiological effect on most types of cells and tissues. Involved in cell migration processes that are specific for lymphocytes. |
| Immunosuppression | Lymphocyte antigen 6 complex, locus H (LY6H) | NM_002347 | [Hs.159590](http://www.ncbi.nlm.nih.gov/UniGene/clust.cgi?ORG=Hs&CID=159590) | 24.7 | 31.4  (=1.3) | 3.8  (↓6.5) | 0.3  (↓74.8) | Lymphocyte recruitment | Highly expressed in brain and lymphoid tissues. Membrane glycoprotein |
| Immunosuppression | Interleukin 12B (IL12B) | NM_002187 | [Hs.674](http://www.ncbi.nlm.nih.gov/UniGene/clust.cgi?ORG=Hs&CID=674) | 6.6 | 5.3  (=1.2) | 1.0  (↓6.6) | 0.1  (↓65.6) | Lymphocyte recruitment | Natural killer cell stimulatory factor , cytotoxic lymphocyte maturation factor |
| Immunosuppression.  Apoptosis inhibition | Granzyme B (granzyme 2, cytotoxic associated serine esterase 1) (GZMB) | NM_004131 | [Hs.1051](http://www.ncbi.nlm.nih.gov/UniGene/clust.cgi?ORG=Hs&CID=1051) | 4.9 | 2.8  (=1.8) | 2.9  (=1.7) | 0.1  (↓48.6) | Inflammation  Apoptosis | Necessary for target cell lysis in cell-mediated immune responses. Linked to an activation cascade of caspases (aspartate-specific cysteine proteases) responsible for apoptosis execution. Cleaves caspase-3, -7, -9 and 10 to give rise to active enzymes mediating apoptosis. |

°FU, fluorescence units

*expressed in neural tissue
